# Supplementary material for: Membrane-bound O-acyltransferase 7 (MBOAT7) shapes lysosomal lipid homeostasis and function to control alcohol-associated liver injury
Source: eLife. 2024 Apr 22;12:RP92243. doi: 10.7554/eLife.92243 (PMC11034944; doi:10.7554/eLife.92243)
Supplement: Figure 1—source data 1. [file elife-92243-fig1-data1.docx]

DASH MBOAT7 Clinical Demographics for Healthy Control vs Heavy Drinkers

|  | | | |  |
| --- | --- | --- | --- | --- |
|  | **Healthy Control (N=25)** | **Heavy Drinker (N=21)** | **Total (N=46)** | **p value** |
| **Sex** |  |  |  | 0.88 |
| Female | 16 (64.0%) | 13(61.9%) | 29 (63.0%) |  |
| Male | 9 (36.0%) | 8(38.1%) | 17 (37.0%) |  |
| **Age** |  |  |  | 0.85 |
| Mean | 45.16 | 44.38 | 44.80 |  |
| Range  **Race** | 23.00 - 63.00 | 21.00 - 63.00 | 21.00 - 63.00 | 0.06 |
| African American | 5 (20.0%) | 13(61.9%) | 18 (39.1%) |  |
| Asian | 1 (4.0%) | 0 (0.0%) | 1 (2.2%) |  |
| Hispanic | 1 (4.0%) | 1 (4.8%) | 2 (4.3%) |  |
| Unknown | 3 (12.0%) | 1 (4.8%) | 4 (8.7%) |  |
| White | 15 (60.0%) | 6 (28.6%) | 21 (45.7%) |  |
| **BMI** |  |  |  | 0.03 |
| Mean | 27.12 | 23.83 | 24.74 |  |
| Range | 22.12 - 30.90 | 15.16 - 28.59 | 15.16-30.90 |  |
| **Bilirubin** |  |  |  | 0.14 |
| Mean | 0.57 | 0.79 | 0.72 |  |
| Range | 0.20 - 0.90 | 0.10 - 1.60 | 0.10 - 1.60 |  |
| **AST** |  |  |  | 0.51 |
| Mean | 24.56 | 26.52 | 25.93 |  |
| Range | 16.00 - 38.00 | 15.00 - 44.00 | 15.00 - 44.00 |  |
| **ALT** |  |  |  | 0.98 |
| Mean | 23.22 | 23.10 | 23.13 |  |
| Range | 12.00 -41.00 | 10.00 - 59.00 | 10.00 - 59.00 |  |
| **Creatine** |  |  |  | 0.94 |
| Mean | 0.91 | 0.90 | 0.90 |  |
| Range | 0.51 - 1.20 | 0.41 - 1.68 | 0.41 - 1.68 |  |
| **INR** |  |  |  | 0.90 |

0.13

| Mean | 1.06 | 1.05 | 1.05 |
| --- | --- | --- | --- |
| Range | 1.00-1.14 | 0.96 - 1.13 | 0.96-1.14 |
| **Albumin** |  |  |  |
| Mean  Range | 3.41  0.51 -4.70 | 4.03  3.50 -4.70 | 3.86  0.51 - 4.70 |

| **Total Protein** |  |  |  | 0.97 |
| --- | --- | --- | --- | --- |
| Mean | 6.90 | 6.91 | 6.91 |  |
| Range  **Globulin** | 6.20 - 7.30 | 5.50 - 8.30 | 5.50 - 8.30 | < 0.01 |
| Mean | 13.50 | 5.48 | 7.56 |  |
| Range | 9.80 - 16.00 | 1.80 - 14.40 | 1.80 - 16.00 |  |
