# Supplementary figures and images for: Membrane-bound O-acyltransferase 7 (MBOAT7) shapes lysosomal lipid homeostasis and function to control alcohol-associated liver injury
Source: eLife. 2024 Apr 22;12:RP92243. doi: 10.7554/eLife.92243 (PMC11034944; doi:10.7554/eLife.92243)

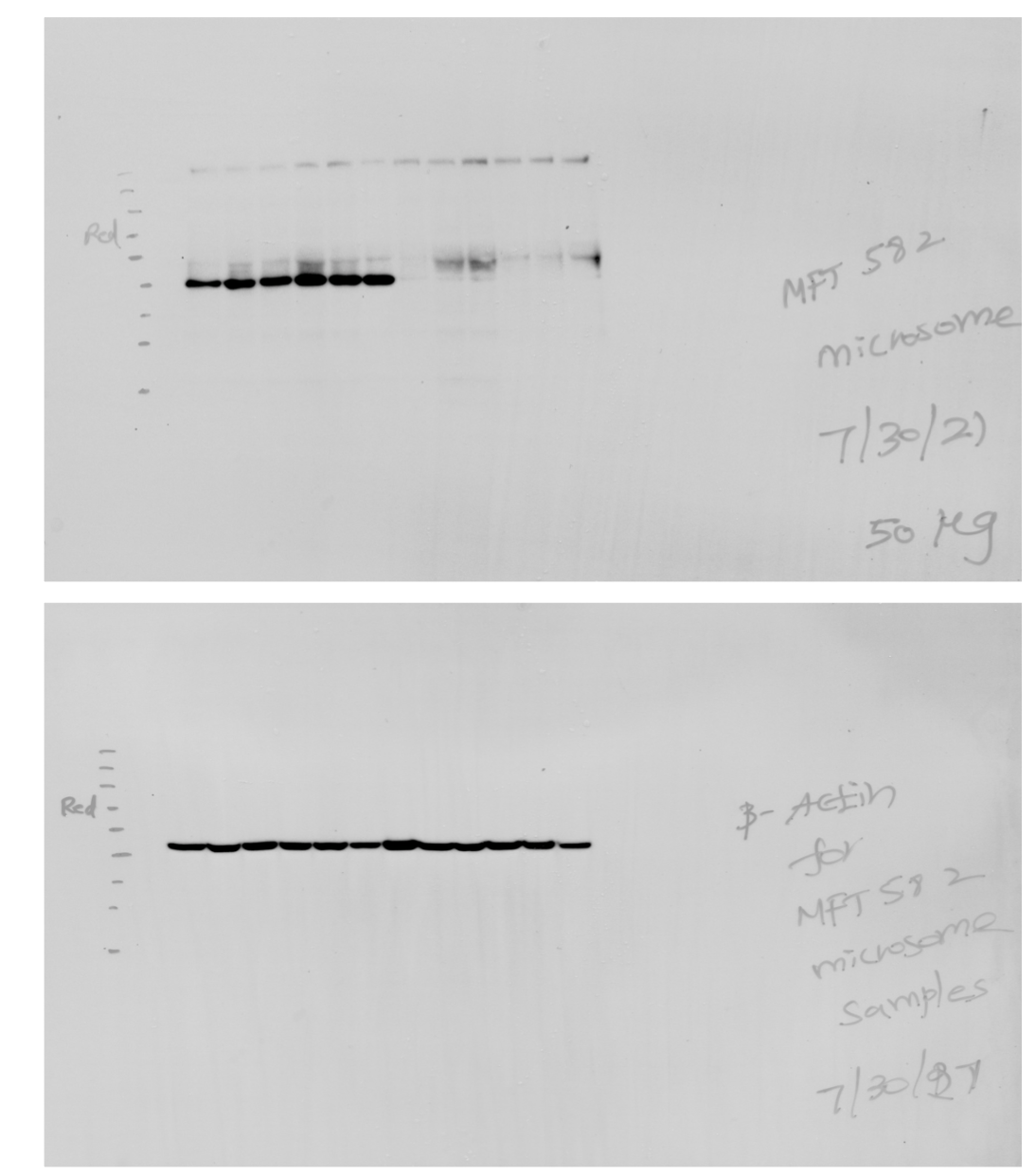

Supplement: Figure 2—source data 1. [file elife-92243-fig2-data1.zip › Fig. 2 B source data 1.tiff]

Figure 2

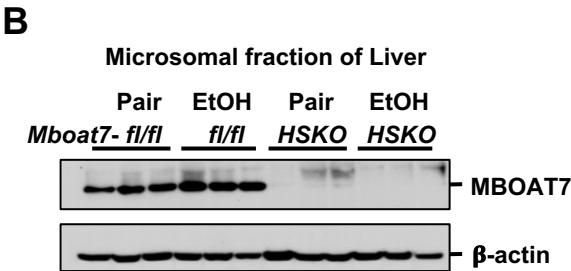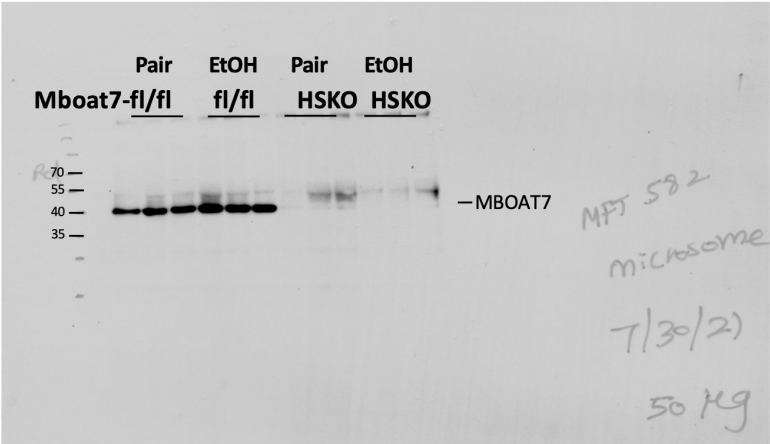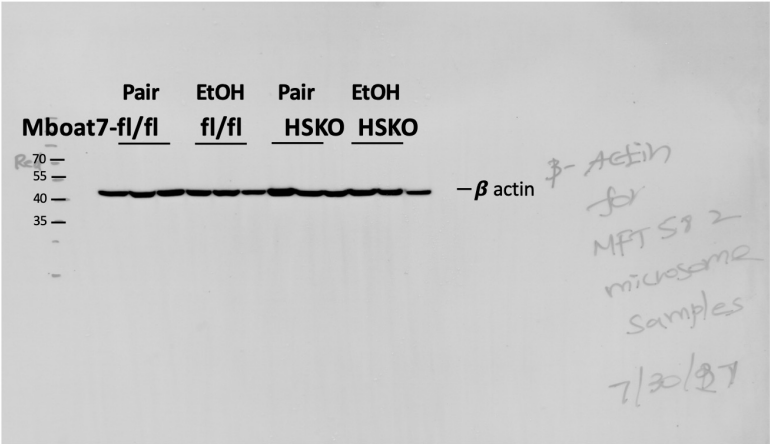

Supplement: Figure 2—source data 2. [file elife-92243-fig2-data2.zip › Fig.2 B source data 2.pdf]

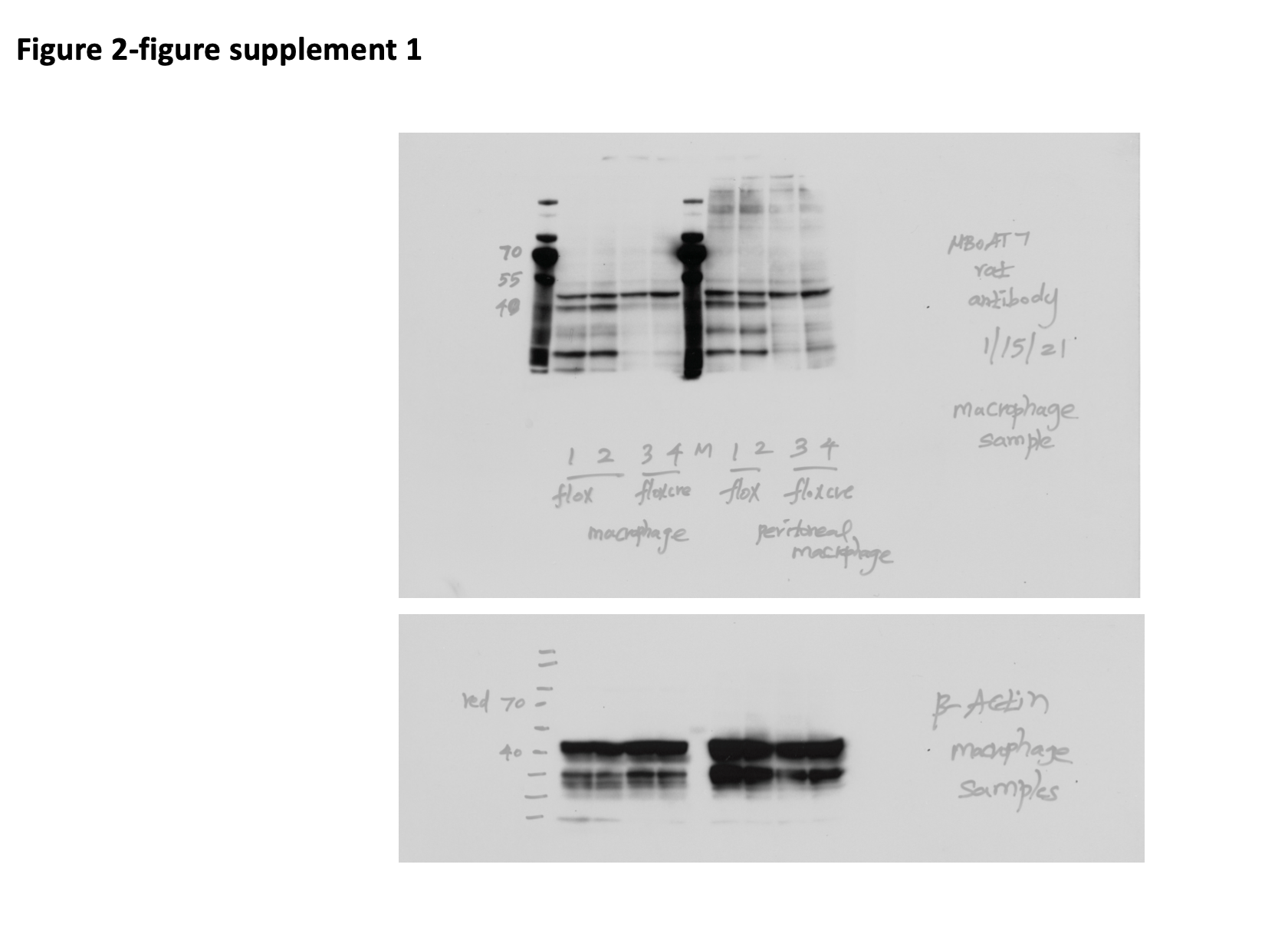

Supplement: Figure 2—figure supplement 1—source data 1. [file elife-92243-fig2-figsupp1-data1.zip › Figure 2-figure supplement 1 source data 1.tiff]

Figure 2-figure supplement 1

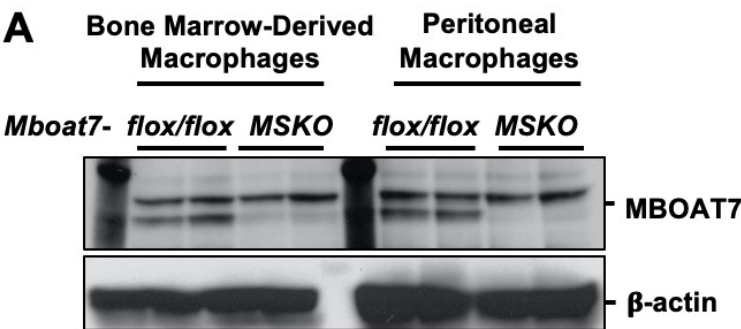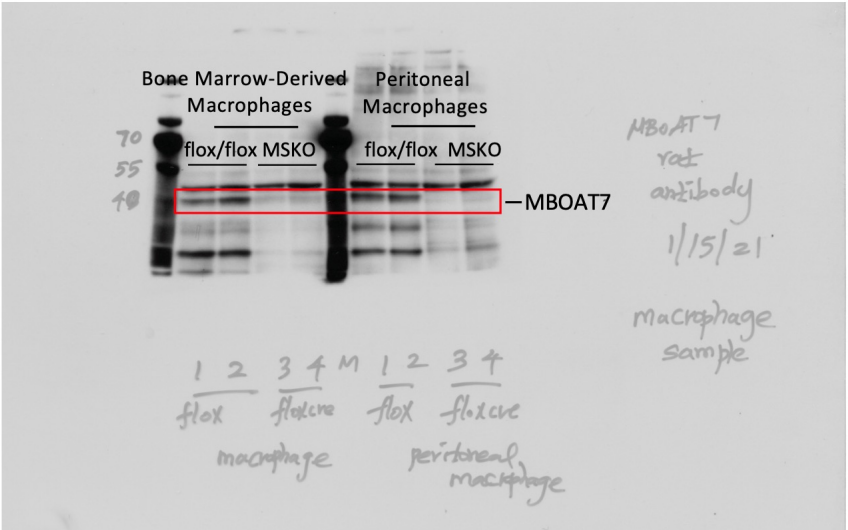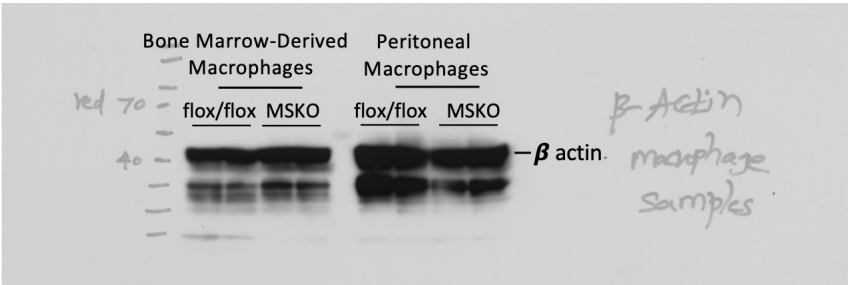

Supplement: Figure 2—figure supplement 1—source data 2. [file elife-92243-fig2-figsupp1-data2.zip › Fig.2-fig supplement 1 A source data 2.pdf]

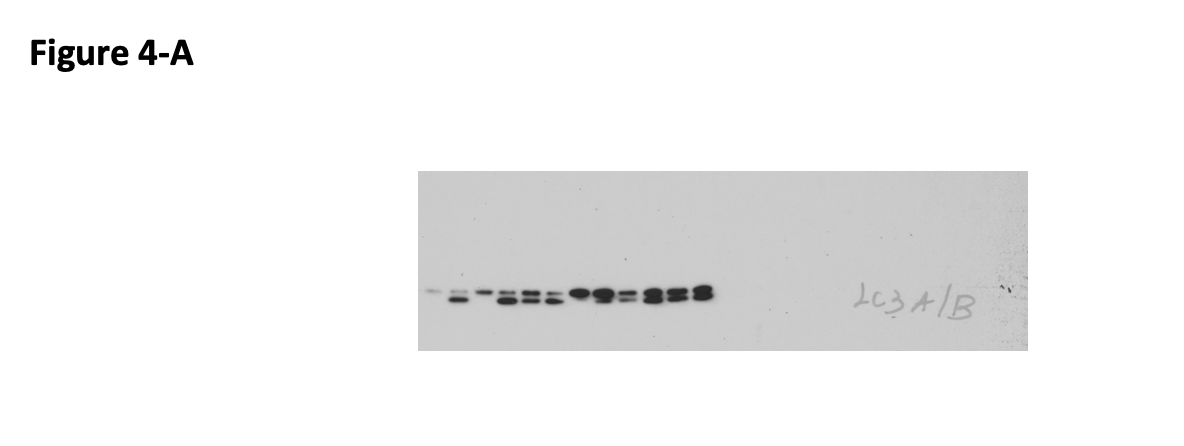

Supplement: Figure 4—source data 1. [file elife-92243-fig4-data1.zip › Figure 4A-source data 1.tiff]

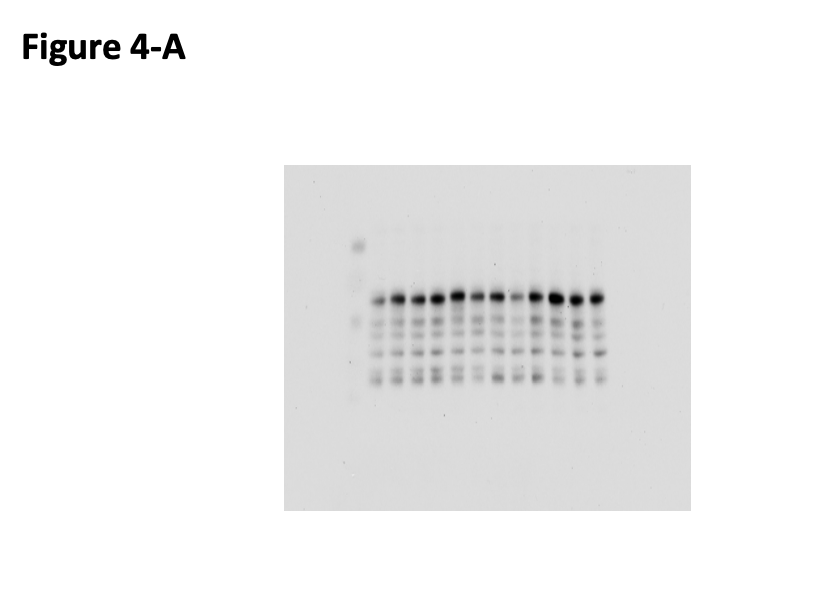

Supplement: Figure 4—source data 2. [file elife-92243-fig4-data2.zip › Figure 4A-source data 2.tiff]

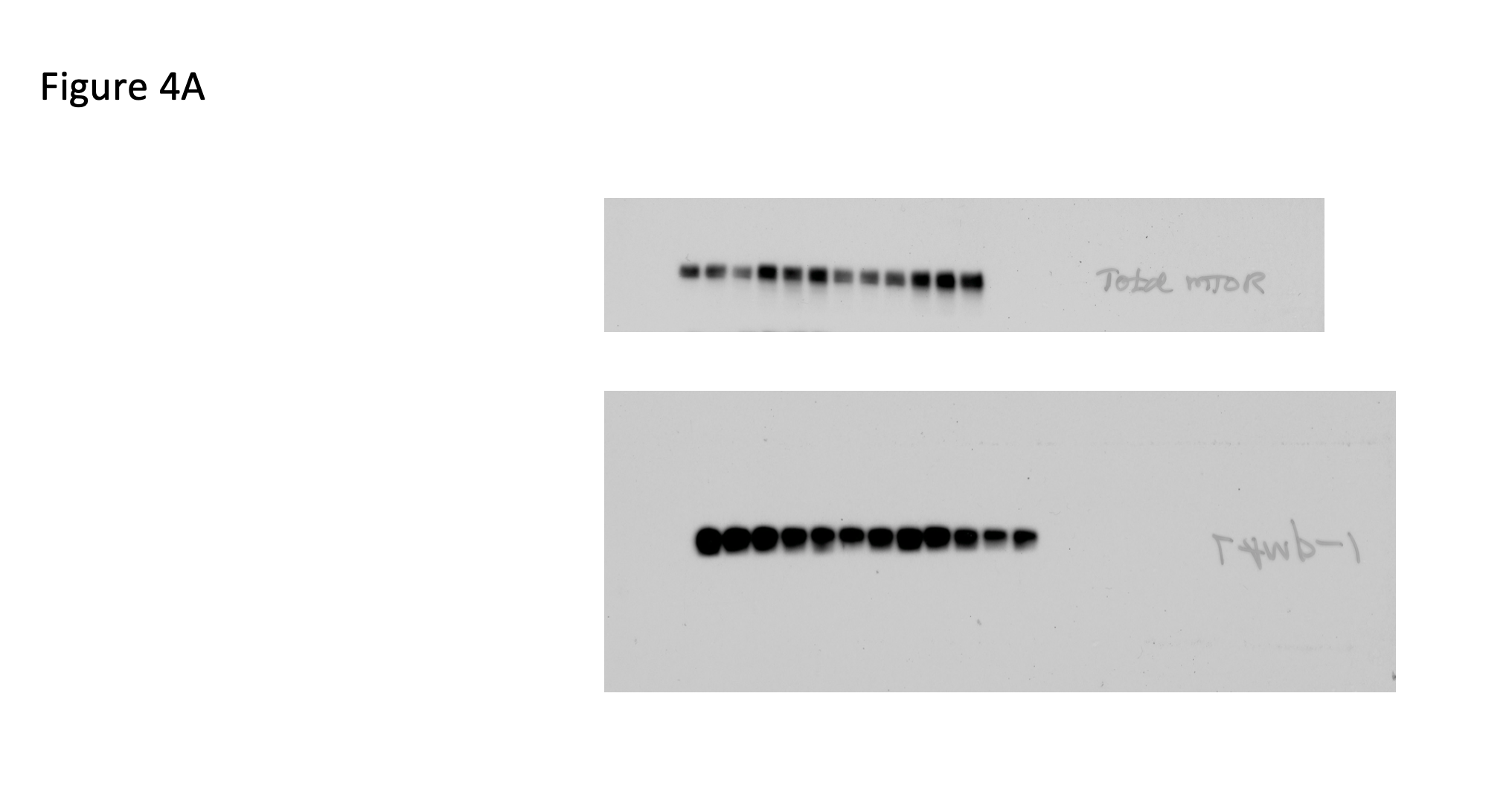

Supplement: Figure 4—source data 3. [file elife-92243-fig4-data3.zip › Figure 4A-source data 3.tiff]

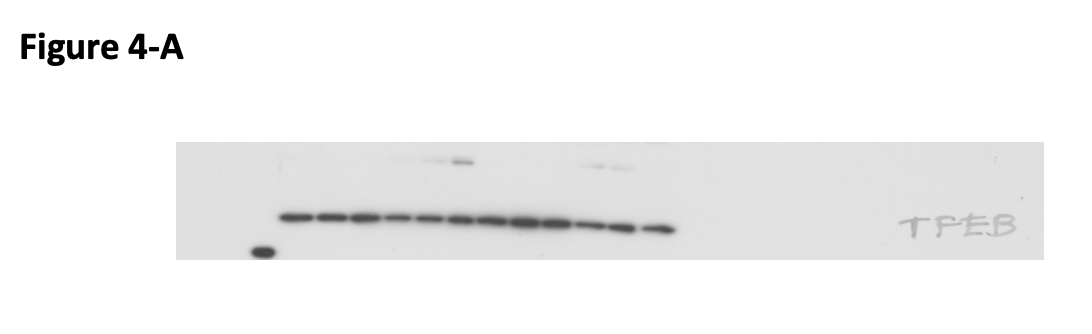

Supplement: Figure 4—source data 4. [file elife-92243-fig4-data4.zip › Figure 4A-source data 4.tiff]

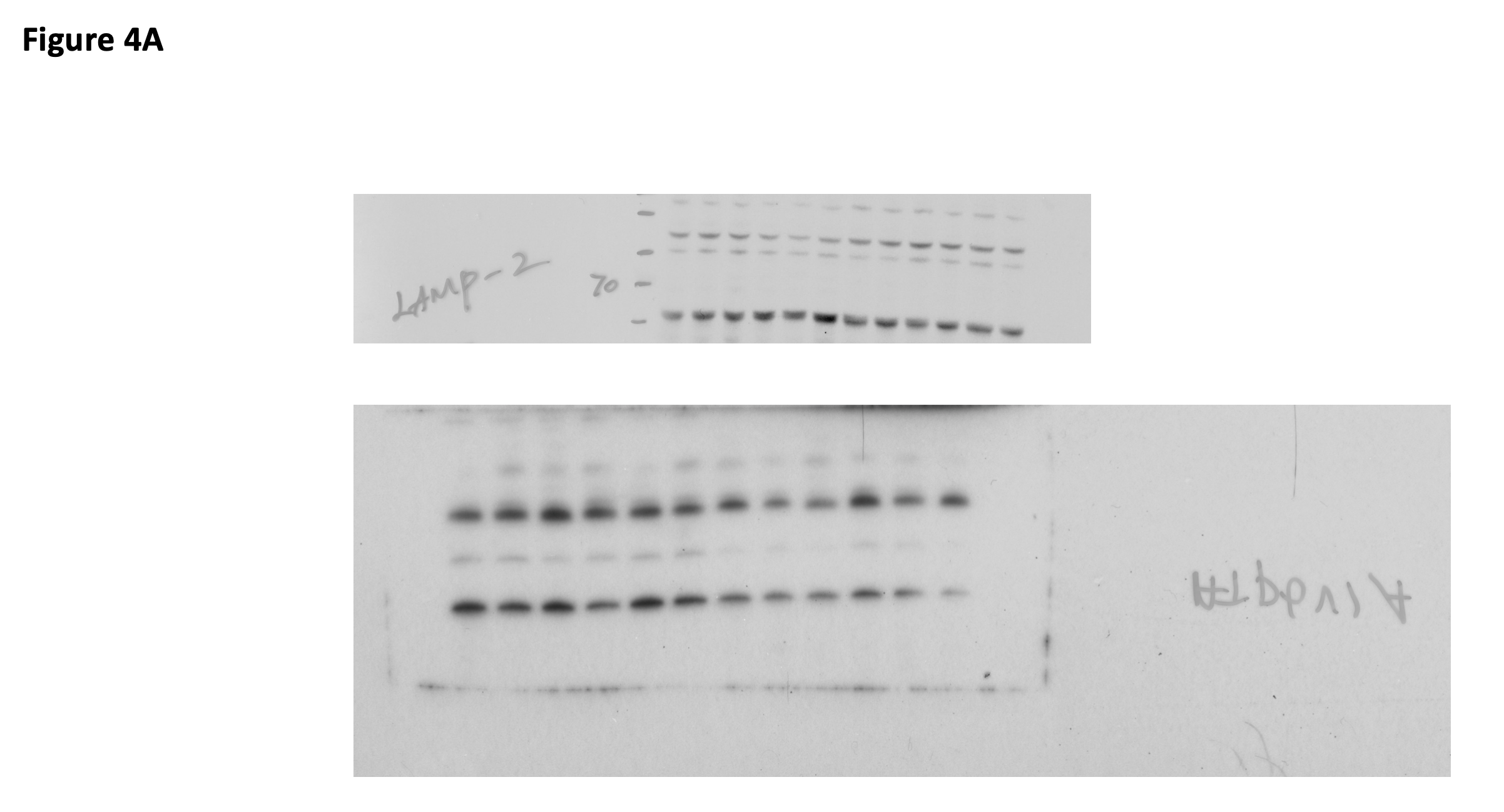

Supplement: Figure 4—source data 5. [file elife-92243-fig4-data5.zip › Figure 4A-source data 5.tiff]

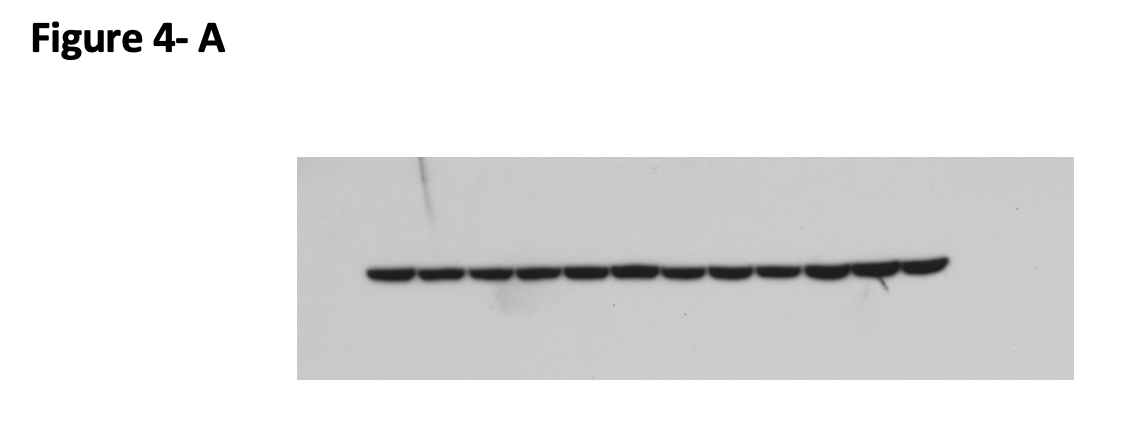

Supplement: Figure 4—source data 6. [file elife-92243-fig4-data6.zip › Figure 4A-source data 6.tiff]

**A**

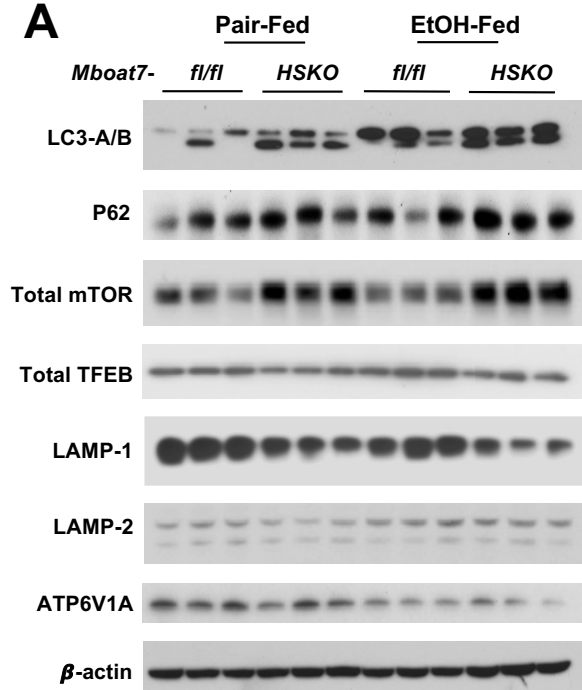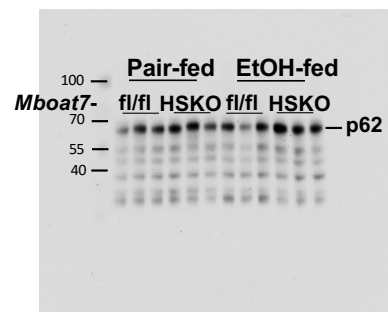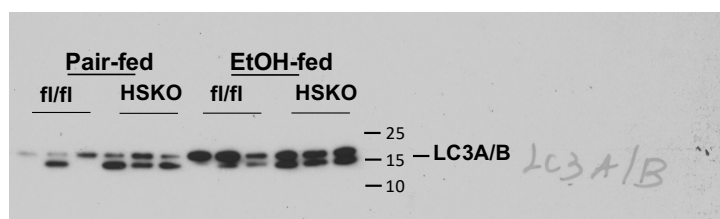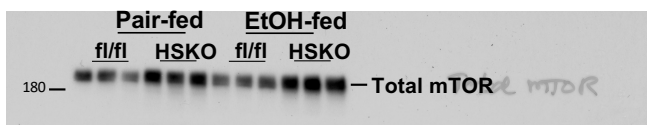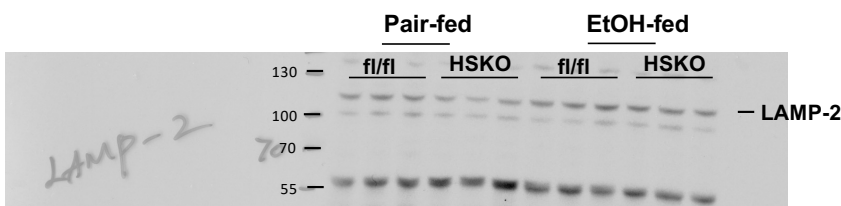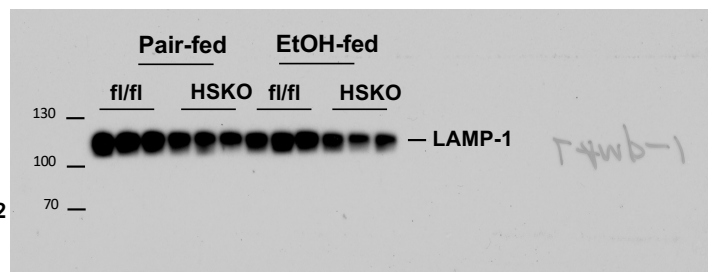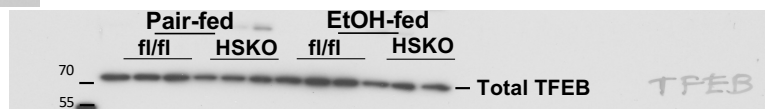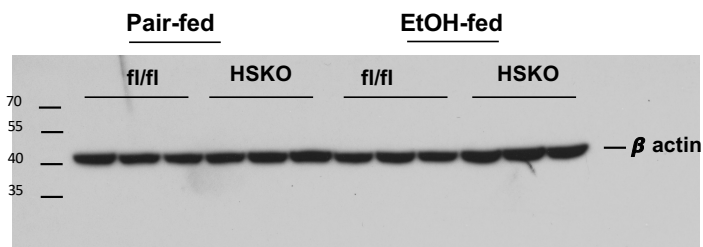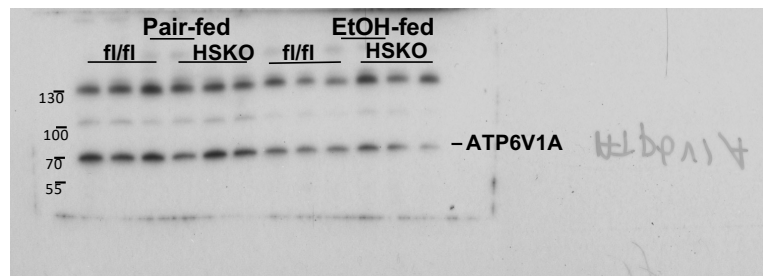

Supplement: Figure 4—source data 7. [file elife-92243-fig4-data7.zip › Figure 4A-source data 7.pdf]

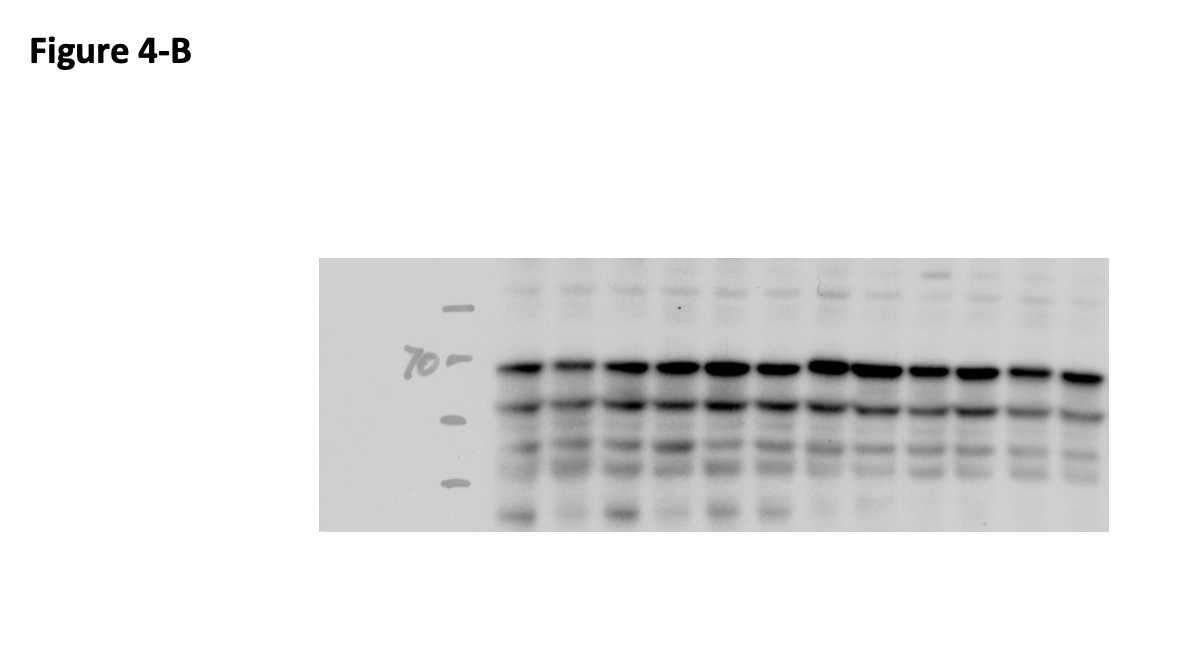

Supplement: Figure 4—source data 8. [file elife-92243-fig4-data8.zip › Figure 4B-source data 8.tiff]

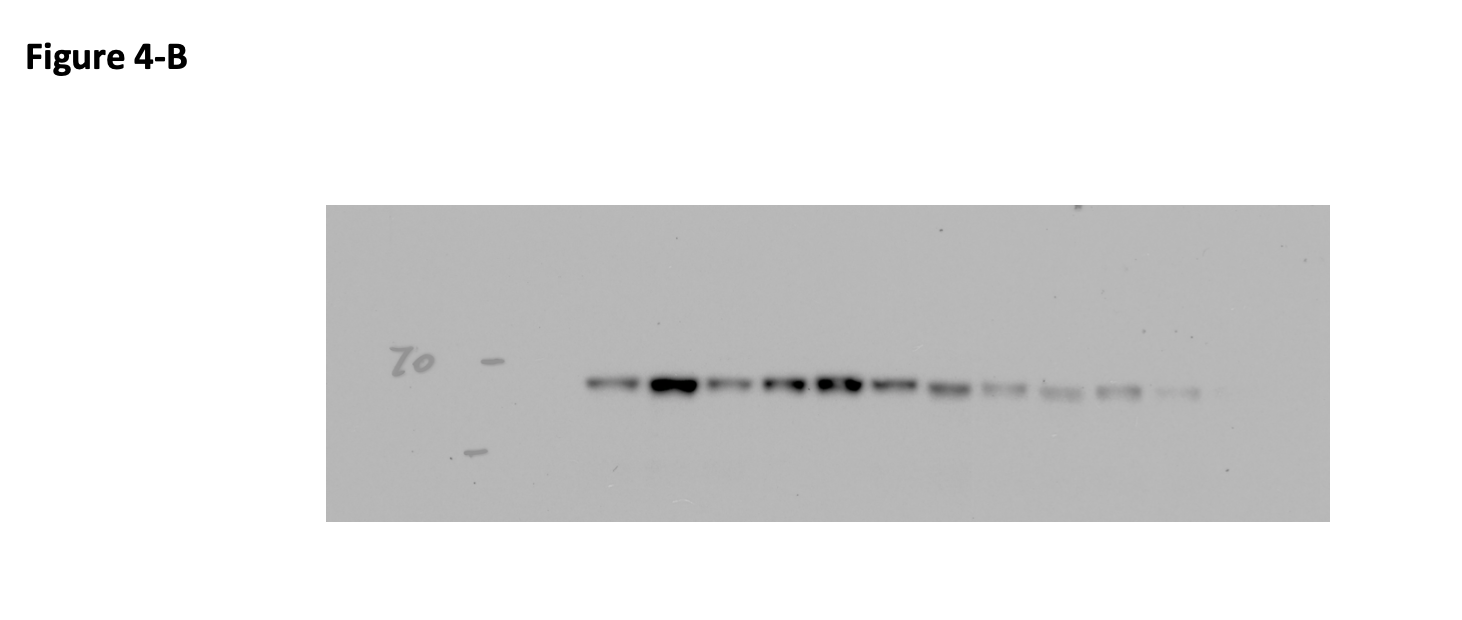

Supplement: Figure 4—source data 9. [file elife-92243-fig4-data9.zip › Figure 4B-source data 9.tiff]

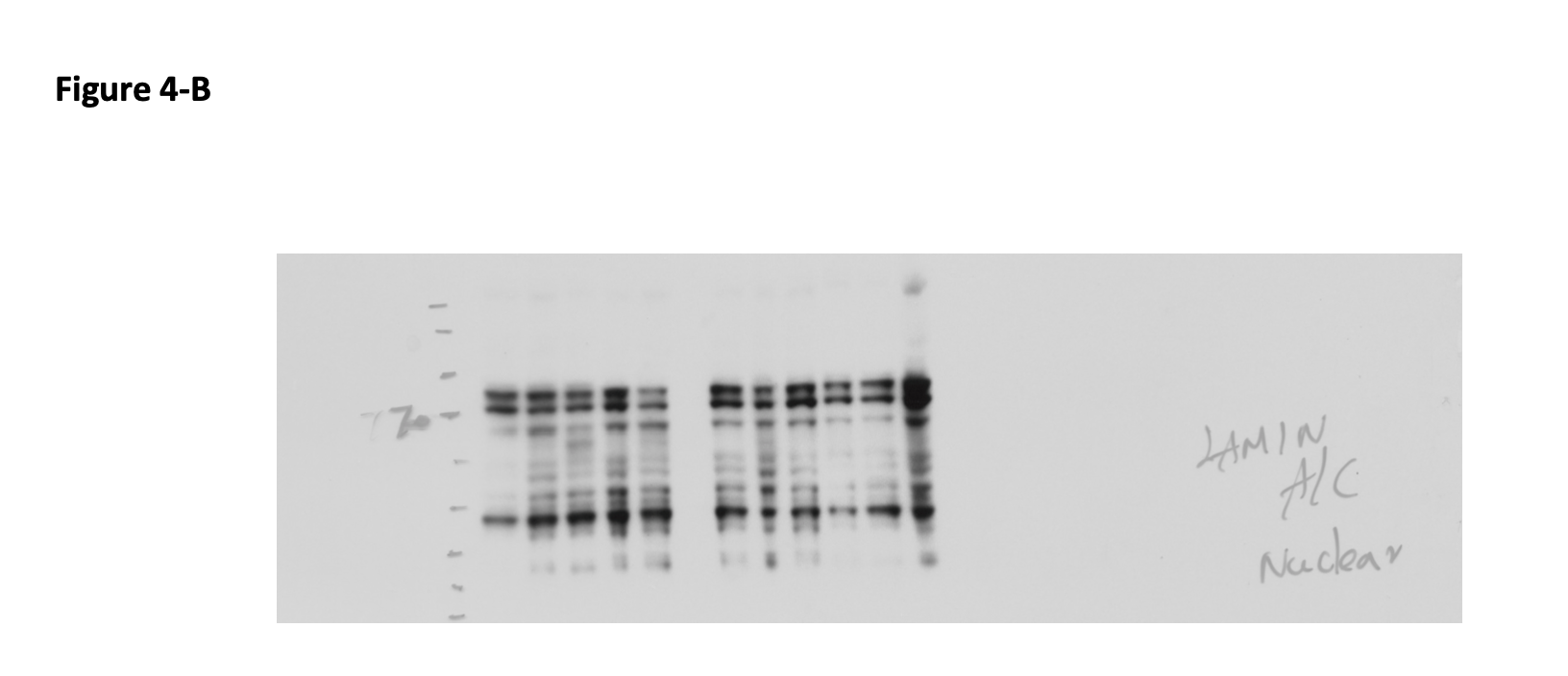

Supplement: Figure 4—source data 10. [file elife-92243-fig4-data10.zip › Figure 4B-source data 10.tiff]

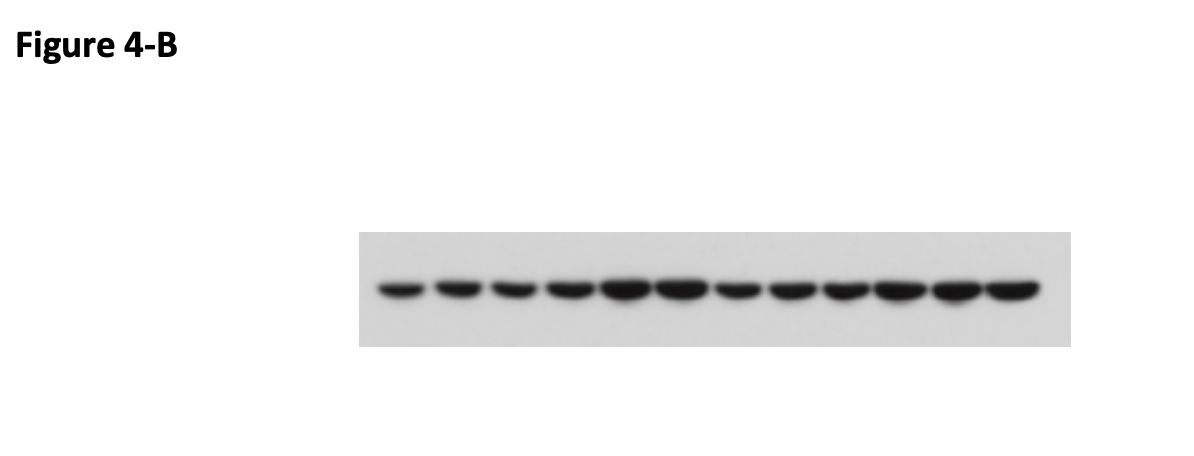

Supplement: Figure 4—source data 11. [file elife-92243-fig4-data11.zip › Figure 4B-source data 11.tiff]

**Figure 4**

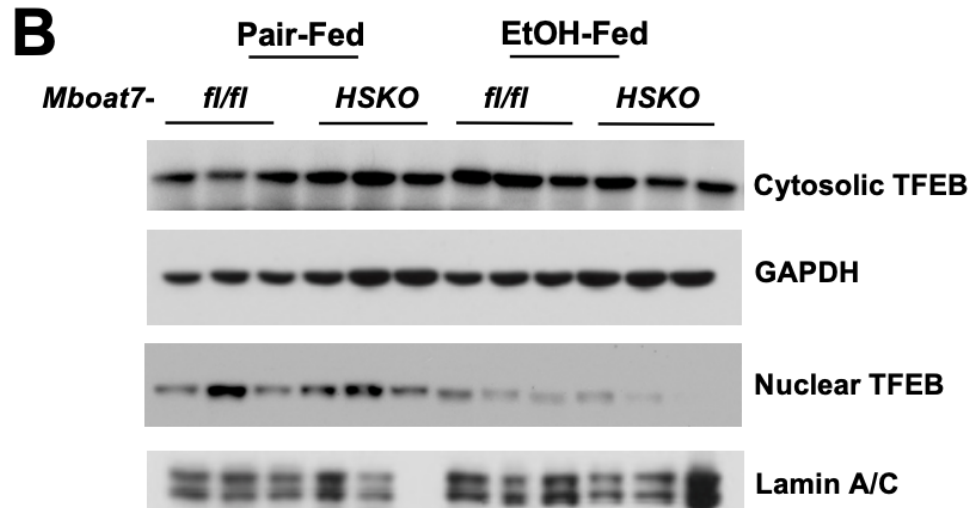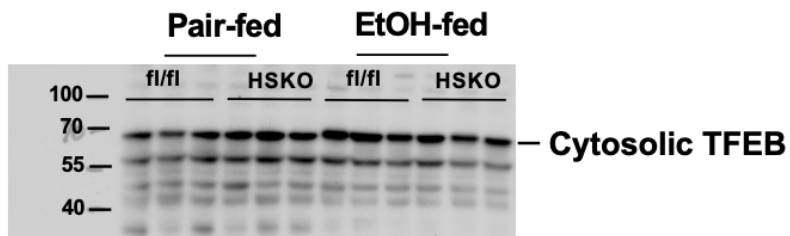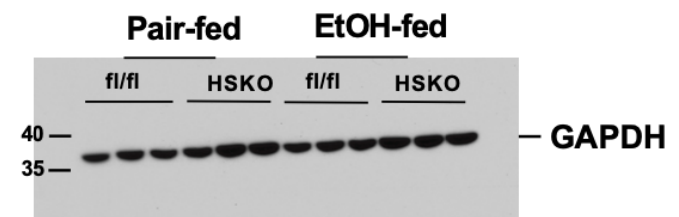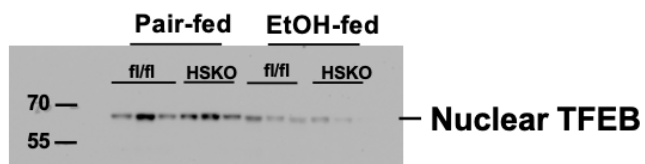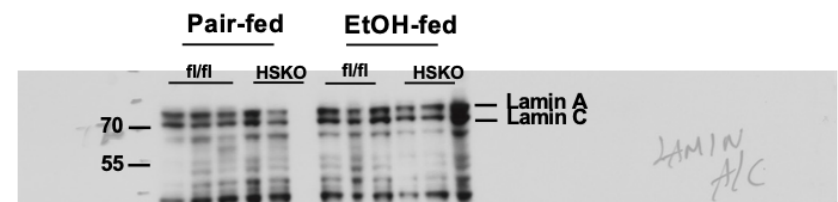

Supplement: Figure 4—source data 12. [file elife-92243-fig4-data12.zip › Figure 4 B-source data 12.pdf]

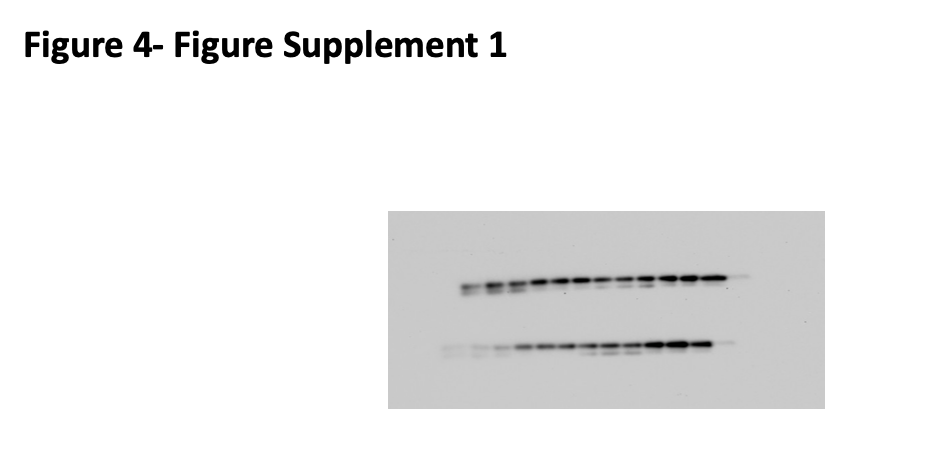

Supplement: Figure 4—figure supplement 1—source data 1. [file elife-92243-fig4-figsupp1-data1.zip › Figure 4-figure supplement 1-source data 1.tiff]

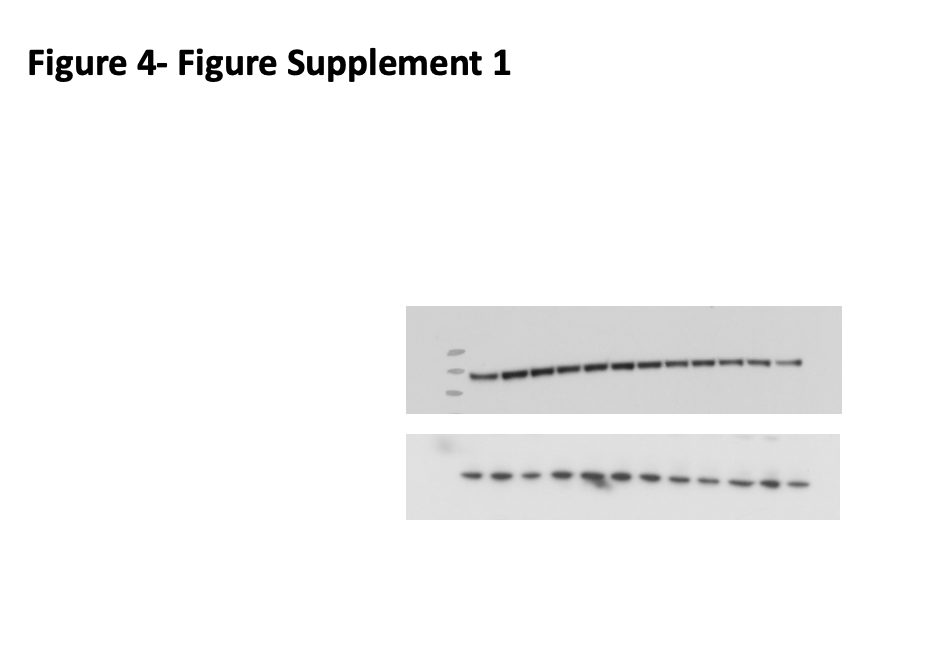

Supplement: Figure 4—figure supplement 1—source data 2. [file elife-92243-fig4-figsupp1-data2.zip › Figure 4-figure supplement 1-source data 2.tiff]

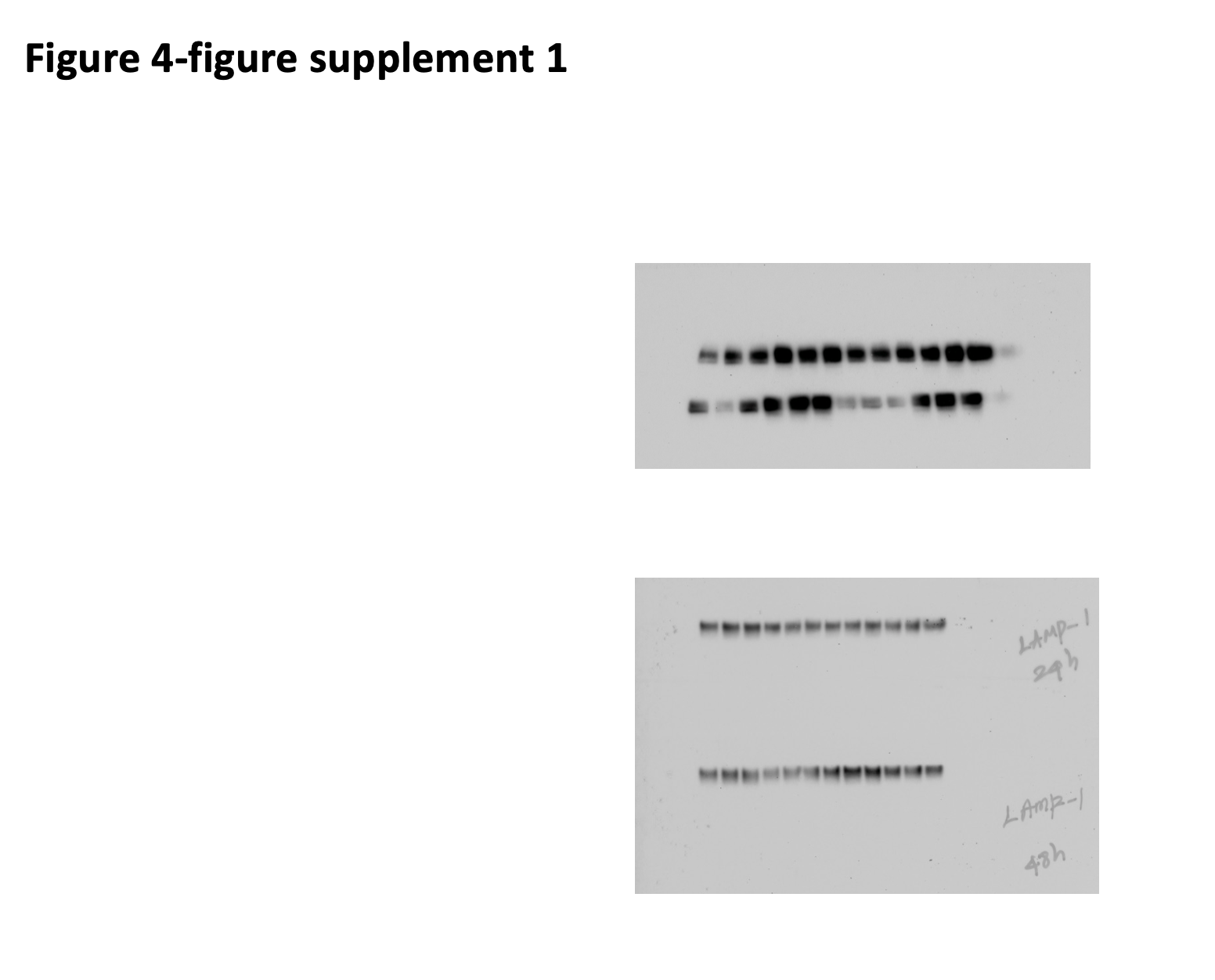

Supplement: Figure 4—figure supplement 1—source data 3. [file elife-92243-fig4-figsupp1-data3.zip › Figure 4-figure supplement 1-source data 3.tiff]

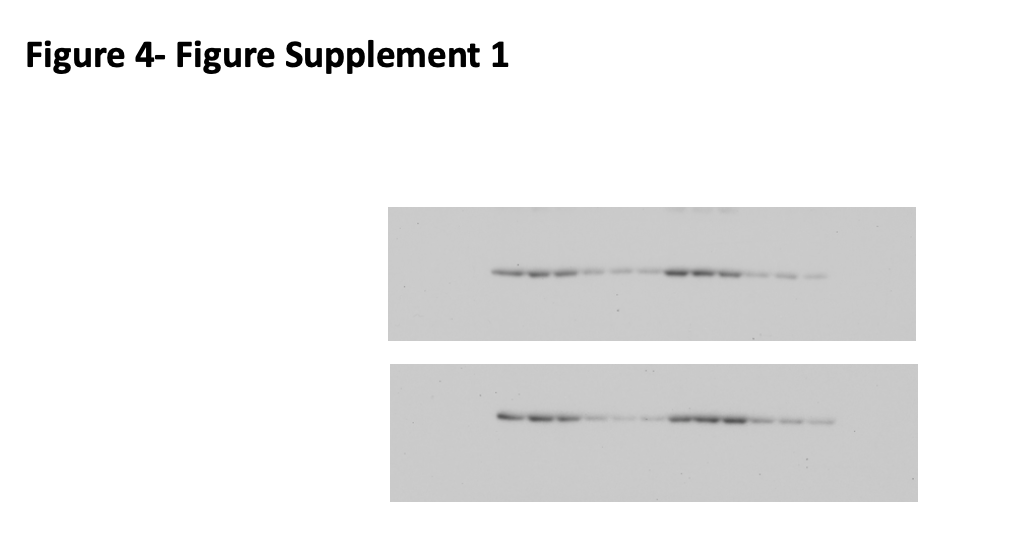

Supplement: Figure 4—figure supplement 1—source data 4. [file elife-92243-fig4-figsupp1-data4.zip › Figure 4-figure supplement 1-source data 4.tiff]

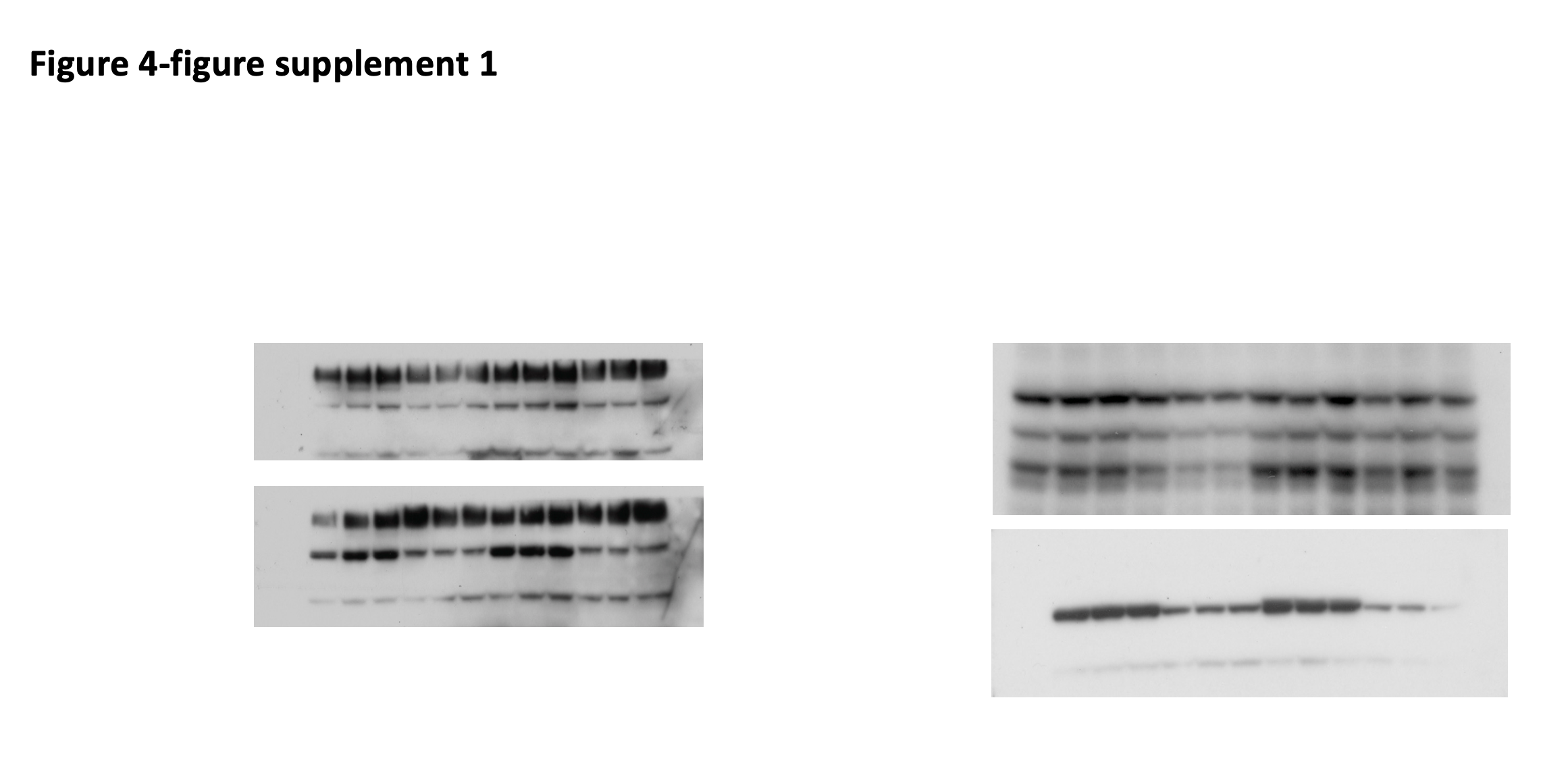

Supplement: Figure 4—figure supplement 1—source data 5. [file elife-92243-fig4-figsupp1-data5.zip › Figure 4-figure supplement 1-source data 5.tiff]

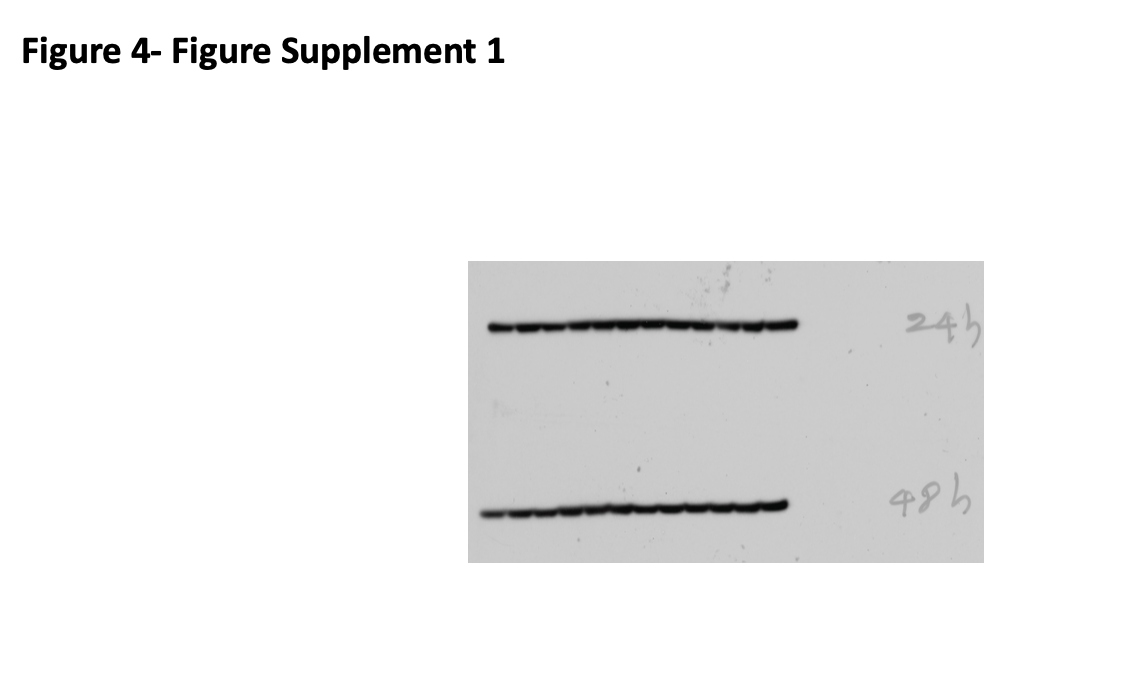

Supplement: Figure 4—figure supplement 1—source data 6. [file elife-92243-fig4-figsupp1-data6.zip › Figure 4-figure supplement 1-source data 6.tiff]

Figure 4-Figure Supplement 1

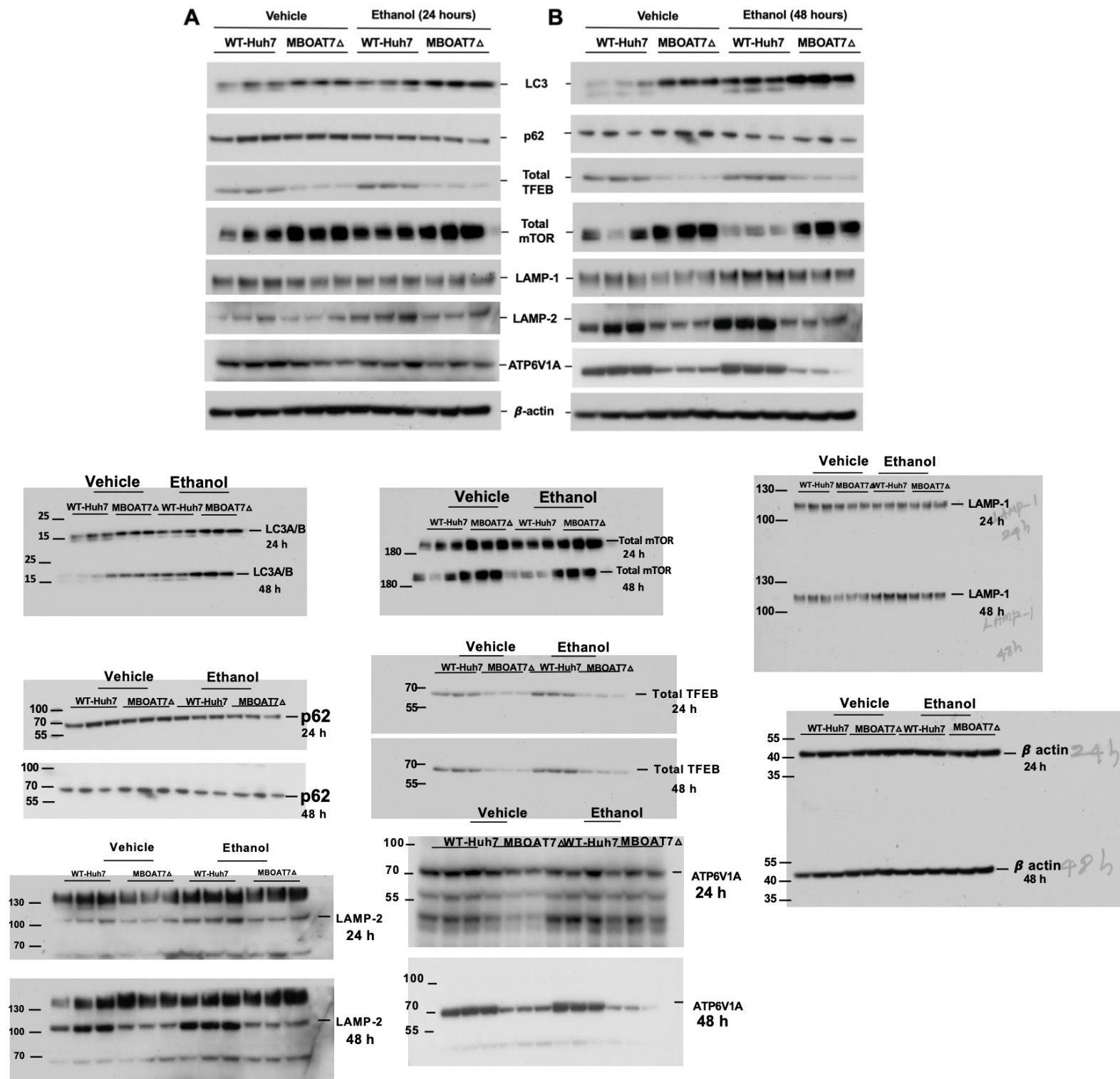

Supplement: Figure 4—figure supplement 1—source data 7. [file elife-92243-fig4-figsupp1-data7.zip › Figure 4 Figure Supplement 1-source data 7.pdf]
