# Supplementary material for: Membrane-bound O-acyltransferase 7 (MBOAT7) shapes lysosomal lipid homeostasis and function to control alcohol-associated liver injury
Source: eLife. 2024 Apr 22;12:RP92243. doi: 10.7554/eLife.92243 (PMC11034944; doi:10.7554/eLife.92243)
Supplement: Supplementary file 1. [file elife-92243-supp1.docx]

| **Primer information** | | | | |
| --- | --- | --- | --- | --- |
| Reagent type (species) or resource | Designation | Source or Reference | Identifiers | Additional Information |
| Sequence-Based Reagent | *Mboat7*_F | This paper | PCR primers | CATGCGGTACTGGAACATGA |
| Sequence-Based Reagent | *Mboat7*_R | This paper | PCR primers | CCAGTAGGCGCTCAGCAG |
| Sequence-Based Reagent | *Cyclophilin A*_F | This paper | PCR primers | GCGGCAGGTCCATCTACG |
| Sequence-Based Reagent | *Cyclophilin A*_R | This paper | PCR primers | GCCATCCAGCCATTCAGTC |
| Sequence-Based Reagent | *Tnfa*_F | This paper | PCR primers | CCACCACGCTCTTCTGTCTAC |
| Sequence-Based Reagent | *Tnfa*_R | This paper | PCR primers | AGGGTCTGGGCCATAGAACT |
| Sequence-Based Reagent | *Il1b*_F | This paper | PCR primers | AGTTGACGGACCCCAAAAG |
| Sequence-Based Reagent | *Il1b*_R | This paper | PCR primers | AGCTGGATGCTCTCATCAGG |
| Sequence-Based Reagent | *Tgfb*_F | This paper | PCR primers | TGGAGCAACATGTGGAACTC |
| Sequence-Based Reagent | *Tgfb*_R | This paper | PCR primers | CAGCAGCCGGTTACCAAG |
| Sequence-Based Reagent | *Il6*_F | This paper | PCR primers | GCTACCAAACTGGATATAATCAGGA |
| Sequence-Based Reagent | *Il6*_R | This paper | PCR primers | CCAGGTAGCTATGGTACTCCAGAA |
| Sequence-Based Reagent | *Mcp1*_F | This paper | PCR primers | TTCCTCCACCACCATGCAG |
| Sequence-Based Reagent | *Mcp1*_R | This paper | PCR primers | CCAGCCGGCAACTGTGA |
| Sequence-Based Reagent | *Tfeb*_F | This paper | PCR primers | CCAGAAGCGAGAGCTCACAGAT |
| Sequence-Based Reagent | *Tfeb*_R | This paper | PCR primers | TGTGATTGTCTTTCTTCTGCCG |
| Sequence-Based Reagent | *Lamp1*_F | This paper | PCR primers | AGCATACCGGTGTGTCAGTG |
| Sequence-Based Reagent | *Lamp1*_R | This paper | PCR primers | GTTGGGGAAGGTCCATCCTG- |
| Sequence-Based Reagent | *Lamp2*_F | This paper | PCR primers | TGCAGTGCAGATGAAGACAAC |
| Sequence-Based Reagent | *Lamp2*_R | This paper | PCR primers | GCTATGGGCACAAGGAAGTTG |
| Sequence-Based Reagent | *Ctsa*_F | This paper | PCR primers | GCTACCTCAGAGCATCGGAC |
| Sequence-Based Reagent | *Ctsa*_R | This paper | PCR primers | GTTAAGCCAAAGCACCACGG |
| Sequence-Based Reagent | *Gba*_F | This paper | PCR primers | GCCTCCCAGAAGAAGACACC |
| Sequence-Based Reagent | *Gba*_R | This paper | PCR primers | ATATCCCCTGGCTGACCCTT |
| Sequence-Based Reagent | *Gla*_F | This paper | PCR primers | TTGGGGTCAGAGCA TTGGAC |
| Sequence-Based Reagent | *Gla*_R | This paper | PCR primers | AGTCATAACCTGCATCCCGC |
| Sequence-Based Reagent | *Psap*_F | This paper | PCR primers | CAGCAGA TGGTCTGGAGCAA |
| Sequence-Based Reagent | *Psap*_R | This paper | PCR primers | CAAGTTCCCAGCTTCGGTGA |
| Sequence-Based Reagent | *Clcn7*_F | This paper | PCR primers | CGAGATGCCTATCCACGCTT |
| Sequence-Based Reagent | *Clcn7*_R | This paper | PCR primers | CCCGGAAGAGCTTGAACACT |
| Sequence-Based Reagent | *Mcoln1*_F | This paper | PCR primers | TGGGCCAATGGATCAGCTTT |
| Sequence-Based Reagent | *Mcoln1*_R | This paper | PCR primers | GTTCTTGTAACTGGCGCTGC |
| Sequence-Based Reagent | *Atp6v1h*_F | This paper | PCR primers | AAGACCAGCAGGTTCGCTAC |
| Sequence-Based Reagent | *Atp6v1h*_R | This paper | PCR primers | TGCAGAAAGGCTGAACAGGT |
| Sequence-Based Reagent | *Atp6v1d*_F | This paper | PCR primers | GAGCACAGACTGGTCGAAA |
| Sequence-Based Reagent | *Atp6v1d*_R | This paper | PCR primers | AGCTGTCAGTTCCTTCGTGG |
| Sequence-Based Reagent | *Ulk1*_F | This paper | PCR primers | ACCATTGTCTACCAGTGT |
| Sequence-Based Reagent | *Ulk1*_R | This paper | PCR primers | AGTGTCTTGTTCTTCTCATAA |
| Sequence-Based Reagent | *Atg2b*_F | This paper | PCR primers | CCTCCACTCTCAGAATCA |
| Sequence-Based Reagent | *Atg2b*_R | This paper | PCR primers | GTCCATCACACGAACATAA |
| Sequence-Based Reagent | *Atg3*_F | This paper | PCR primers | TCACAACACAGGTATTACAG |
| Sequence-Based Reagent | *Atg3*_R | This paper | PCR primers | CTTCCTCGTCTTCTTCATC |
| Sequence-Based Reagent | *Atg7*_F | This paper | PCR primers | CAGAAGAAGTTGAACGAGTA |
| Sequence-Based Reagent | *Atg7*_R | This paper | PCR primers | CAGAGTCACCATTGTAGTAAT |
| Sequence-Based Reagent | *Lc3*_F | This paper | PCR primers | GCTCTTTGTTGGTGTGTA |
| Sequence-Based Reagent | *Lc3*_R | This paper | PCR primers | TCTTCTGTTGCTGTTGTC |
